# Evaluation of Dextrose Water, Black Tea and Orange Juice on Histopathologic Recovery of Surgery-Induced Intestinal Damage in Rabbits

**DOI:** 10.5812/traumamon.4781

**Published:** 2012-07-31

**Authors:** Mehrdad Hosseinpour, Hassan Ehteram, Maryam Farhadi, Samin Behdad

**Affiliations:** 1 Trauma Research Center, Kashan University of Medical Sciences, Kashan, IR Iran; 2 Department of Pathology, Kashan University of Medical Sciences, Kashan, IR Iran

**Keywords:** Orange Juice, Black Tea, Dextrose Water, Postoperative Period

## Abstract

**Background:**

The increase in intestinal permeability following damage to the intestinal mucosa in any surgical procedure, trauma or fasting is well- known.

**Objectives:**

Our objective was to experimentally evaluate whether antioxidant consumption is associated with decreased intestinal damage in intestinal surgical procedures.

**Materials and Methods:**

Forty rabbits were used to compare the pathological changes in the intestine after consumption of dextrose water 5% (D5W), black tea and orange juice in fasting and in cases with intestine resection and anastomosis. They were divided in to five groups as shams (GI), NPO (G II), D5W (GIII), black tea (GIV) and orange juice groups (GV). In GII to GV group with median laparotomy, a 2 cm segment of jejunum was resected and ends of the bowel were anastomosed. Postoperatively, animals fasted for five days. Animals in GII were killed after five days of fasting. On day five case groups were given free access to drinking D5w (GIII), black tea solution (G IV) and orange juice (GV) for a further 7 days. On day 8, animals were reoperated and the repaired segment of intestine was removed. Morphologic data were compared in groups.

**Results:**

There were 8 rabbits in each group. There was a significant difference in villi lengths in the groups (P = 0.003). GV rabbits had obvious recovery of the villous architecture.

**Conclusions:**

Orange juice as a source of vitamin C may be an appropriate liquid for postoperative recovery following intestinal surgery.

## 1. Background

The increase in intestinal permeability is well-known following damage to the intestinal mucosa in any surgical procedure, trauma or fasting (1-3). Oxidative stress on enterocytes has been shown to be intimately associated with this response. Abdeen and colleagues (1) recently demonstrated that use of green tea and vitamin E significantly reduced fasting–induced mucosal damage in rat intestine. While their data was obtained from fasted rats, this article has been triggered by recent reports on the potential negative effects of local increase in superoxide radicals on the injured intestinal mucosa (4, 5).

Elevated local superoxide radicals in the villi and crypts were found to be a significant mediator for protease activation after surgical stress in the intestine (6). In an experimental study on 42 unfed guinea pigs, Gonul (7) reported that ascorbic acid (vitamin C) supplementation is associated with protection of plasma membrane by removing the free radicals. All of these previous mentioned studies evaluated the effects of antioxidants in fasting and/or intestinal manipulation.

## 2. Objectives

Our objectives in this study were to experimentally evaluate whether antioxidant consumption is associated with decreased intestinal damage in intestinal surgical procedures such as intestinal resection and anastomosis. The second objective of this study was to choose a suitable liquid for starting the post-operative regimen in these cases.

## 3. Materials and Methods 

After the ethics committee approved the study. 40 rabbits were used in our study to compare the pathological changes in the intestine after consumption of dextrose water 5% (D5W), black tea and orange juice in fasting and in cases with intestine resection and anastomosis. White, male, adult New Zealand rabbits (Razi institute, Tehran, Iran) weighting 1500-2800 g identified by numbering from 1 to 40, tattooed on the internal face of the right ear were used. The rabbits were kept in a controlled environment (temperature: 24-26 °C, humidity: 55-65%), fed on a commercial pellet diet (Niro–Sahand Co, Tabriz, Iran) and allowed free access to tap water until four hours before surgery when feeding was discontinued.

They were randomly divided in to five groups:

Group I (GI): 8 animals as shams.

Group II (GII): 8 animals fasted for five days (control group).

Group II (GIII): 8 animals with surgical procedure and D5W regimen.

Group IV (GIV): 8 animals with surgical procedure and black tea regimen.

Group V (GV): 8 animals with surgical procedure and orange juice regimen.

Anesthesia was administered via intramuscular pre-medication of 2 mg/kg body weight acepromazine (30 minute before anesthesia) and 4mg/kg body weight xylazine with 40 mg/kg body weight ketamine intramuscularly. The animal was placed in a horizontal dorsal decubitus position on the surgical table and its paws fixed to the extremities of the table with thin ropes. An electrical clipper was used to shave abdominal hairs from the abdomen. Antisepsis of the surgical site was done using 2% iodinated alcohol solution. Median laparotomy was performed starting 1 cm below the processus xiphoides, in caudal direction with 4 cm extension. After exteriorization of intestinal loops, from a distance of 15 cm of Treitz ligament, a 2 cm segment of jejunum was resected and the ends of bowel were anastomosed using Vicryl 0/4 in single layer. The intestine was then placed back into the abdominal cavity. Following this, the abdominal wall was closed and the animals were allowed to recover from anesthesia. Post operatively, animals fasted for five days. During this period, they received 10 ml of dextrose 50%, 15 ml of lipid solution 10%, 10 ml of amino acid solution 5% and 15 cc lactated Ringer electrolyte solution, intraperitoneally daily.

Animals in GI were killed without fasting for baseline morphologic evaluation of rabbit intestine. Animals in GII were killed after five days of fasting. Groups III-V were also fasted as described for GII, but on day five were given free access to drinking D5w (GIII), black tea solution (G IV) and orange juice (GV) for further 7 days. Intraperitoneal total nutrient admixture was continued during this period. For black tea solution preparation, dried leaves of commercially available black tea were purchased locally. The tea was prepared by adding 16 g of dry leaves of tea to 1 L of boiled drinking water.

For orange juice preparation, fresh natural oranges was used. On day 8, animals were anaesthetized and their abdominal wall was re-opened and the repaired segment of intestine was removed. The lumen of the removed intestine was flushed immediately with 10 % formalin, and the specimen was fixed in 10 % formalin for 24 h. Specimens were stained with hematoxylin and eosin for histological examination. For morphologic evaluation, a single–blind technique was used, in which the pathologist, who was responsible for microscopic analysis, was unaware of the allocation of the study cases.

The lengths of the villi were measured by a cross-line micrometer. Other morphologic data were reported qualitatively. These data included goblet cells distribution, inflammatory cells infiltration, villi fragmentation, crypts wideness and subephitelial lymphangectasia. Quantitative data were reported as mean ± SD. Data were checked for normality using the Kolmogorov-Smirnov test. K-sample Kruskal-Wallis test was used to compare the lengths of the villi in groups. P-values < 0.05 were considered significant. Data were analyzed using SPSS (SPSS Inc, version 12).

## 4. Results

The groups were similar in age and sex (male). There were 8 rabbits in each group. All rabbits survived until the end of the experiment. The mean body weight of rabbits was 1284 ± 152.7 gr. There was no significant difference between the groups regarding body weight.

### 4.1. Histopathologic Changes

**Figure 1:**
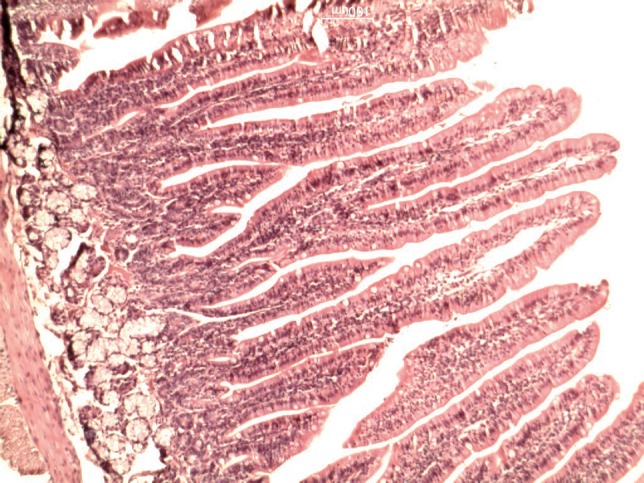
Histologic changes in the intestinal mucosa in shams

**Figure 2:**
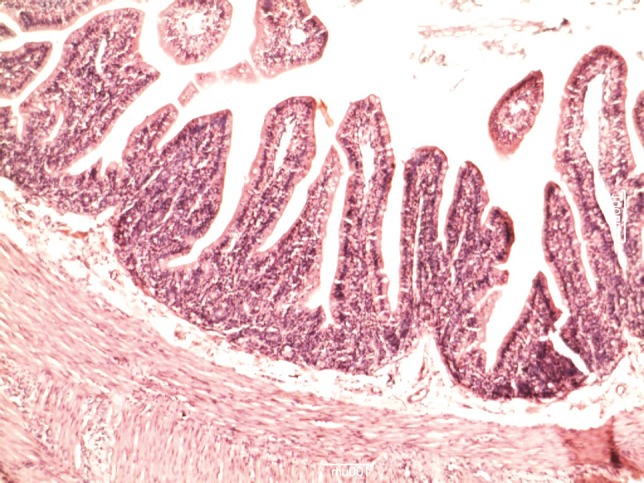
Histologic changes in the intestinal mucosa during the recovery period in GII (NPO)

**Figure 3:**
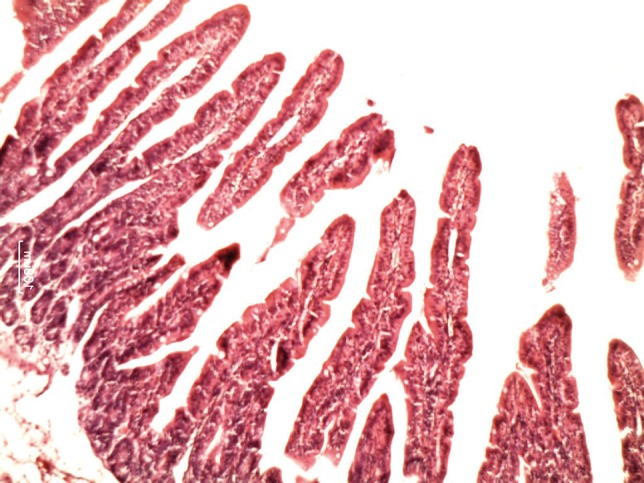
Histologic changes in the intestinal mucosa during the recovery period in GIII (Dextrose Water)

**Figure 4:**
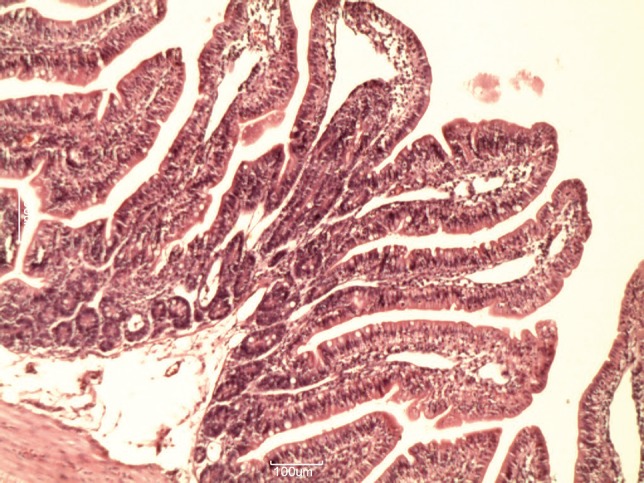
Histologic changes in the intestinal mucosa during the recovery period in GIV (Black Tea)

**Figure 5:**
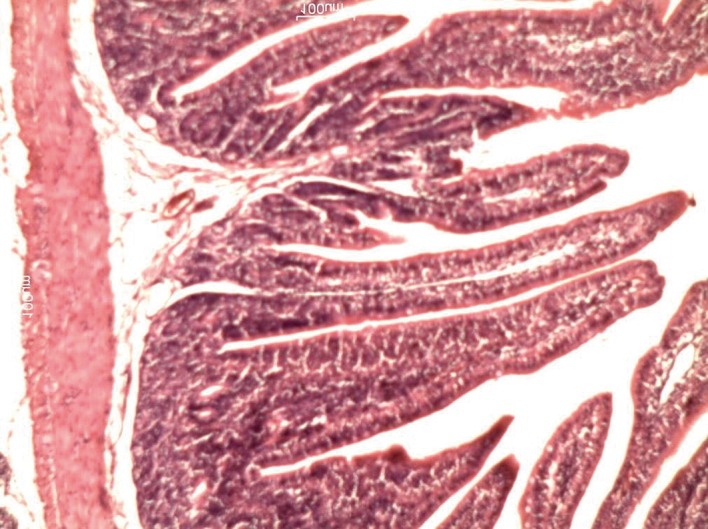
Histologic changes in the intestinal mucosa during the recovery period in GV (Orange Juice)

Table 1 shows the lengths of the villi in the groups. There was a significant difference in villi lengths in the groups (P = 0.003). In comparison with GI (Figure 1), in all of other groups (Figures 2, 3, 4 and 5), the number of goblet cells were decreased. Infiltration of inflammatory cells and lymphangectasia, villi fragmentation and crypt wideness also increased in case and control groups. In comparison to control group (GII) with experimental groups we found that villous changes, inflammatory cells infiltration and goblet cells reduction were more prominent in unfed rabbits. In comparison of case groups, villous change and inflammatory cell infiltration were more prominent in GIII and GIV. On the other hand GV rabbits had obvious recovery of the villous architecture. In comparison of GIII and GIV, we found that inflammatory cells infiltration was more prominent in G4 group.

**Table 1:** Villi Length Measurement in Groups (μm)

	Mean ± SD	Min	Max
Shams	861 ± 35.22	815	900
Controls	335.5 ± 70.33	265	432
D5W a	577 ± 120.41	405	680
Black tea	501.75 ± 94.1	402	623
Orange juice	818 ± 94.15	705	934

^a^Abbreviation: D5W: Dextrose Water 5%

## 5. Discussion

The ability of the intestinal mucosa to maintain nutrient absorption and barrier action against the invasion of luminal bacteria is a crucial survival mechanism. The various oxidative responses that occur after surgical stress, trauma and fasting are classically tough to change intestinal permeability. One such physiologic response is the local increase in superoxide radicals in the villi and crypts of the small intestinal mucosa associated with stress. It is thought to be cause of mitochondrial and cytosolic protease activation (6). Therefore, there has been growing evidence that antioxidants can prevent and improve superoxide radical–induced intestinal injury. A study of five groups of rats in Kuwait University (8) demonstrated a higher total plasma antioxidants with green tea and vitamin E consumption.

However, the majority of studies in this field have been reported in non–intestinal surgery conditions such as fasting or intestine manipulation. In this study, we evaluated the effects of three common postoperative starting regimens (D5W, black tea and orange juice) on recovery of surgical–stress induced intestinal mucosal injury in small bowel resection and anastomosis. Our findings showed that orange juice is a better regimen for postoperative recovery period. Although Asfar et al. (8) showed that green tea has more preventive effects on fasting induced intestinal mucosal injury, in this study; we did not use green tea because its consumption and preparation is limited in our country. The length of villi in rats of Asfar study (8) showed significant differences in pre-treatment groups. Its mean was 4 µm in controls, 3.5 µm in fasted animals, 4.5 µm in black tea group and 6 µm in vitamin E group. As our findings, they showed that vitamin E as an antioxidant can increase the recovery rate of villi length. Gonul et al. showed that vitamin C supplementation protects the plasma membrane by reacting with and removing free radicals (7).

Vitamin C is reported to have antioxidant activity in both in vivo and in vitro studies. It significantly inhibits the production of super oxide and hydrogen peroxide (9). The process of intestinal healing mimics that of wound healing elsewhere in the body in that it can be arbitrarily divided into an acute inflammatory (lag) phase, a proliferative phase, and, finally, a remodeling or maturation phase. Collagen is the single most important molecule for determining intestinal wall strength, which makes its metabolism of particular interest for understanding anastomotic healing. Several factors that are known to inhibit collagen synthesis, such as vitamin C deficiency, zinc deficiency, jaundice, and uremia, have a detrimental effect on tissue healing. A critical stage in collagen formation is the hydroxylation of proline to produce hydroxyproline; this process is believed to be important for maintaining the three-dimensional triple-helix conformation of mature collagen, which gives the molecule its structural strength. Vitamin C deficiency results in impaired hydroxylation of proline and the accumulation of proline-rich, hydroxyproline-poor molecules in intracellular vacuoles.

Our findings regarding black tea are similar to results of Asfar (8). Black tea offered no significant protection on intestinal mucosa in comparison to D5W. On the other hand, although green tea mimicked the effects of antioxidants in intestinal mucosal cells following fasting-induced intestinal damage, but black tea has no each significant effects. One of the major limitations of this study was inability to measure the vitamin C and antioxidant concentrations of study solutions. We also have some limitation to conclude of our findings in human beings. In clinical practice, in most of cases, solid regimen is started in a few days after operation; therefore it seems that in other studies, these effects should be evaluated in shorter course. In conclusion, in this study we suggest that orange juice as a source of vitamin C is a most appropriate liquid for post-operative starting regimen is intestinal surgery.
